# The Physician’s Wife

**Published:** 1894-04

**Authors:** 


THE PHYSICIAN'S WIFE; and The Things that Pertain to Her Life. By Ellen M. Firebaugh. With portrait of author and 44 photo-engravings of original sketches. In one crown octavo volume of 200 pages. Extra cloth, $1.25 net. Special limited edition, first 500 copies, numbered, and printed in photo-gravure ink on extra-fine enamelled paper; bound in half-leather and vellum cloth, $3.00 net. Philadelphia: The F. A. Davis Co., Publishers, 1914 and 1916 Cherry Street.

This charming and clever work, inscribed to Mrs. Frances Hodgson Burnett by the author, was written first to be read at a society meeting. It so pleased the society that its publication was decided upon—and later reached the size it now comes to us. It is well written, full of pleasing incidents, illustrated with 44 photo-engravings, and ends with a beautiful legend. What annoyances a physicians wife has to endure will be appreciated by those who have tried this life, and our non-professional friends will understand what a doctor's life is, and how many trials, worriments, and even persecutions he has to endure, many of which are not mentioned. An hour will not be wasted spent with this little volume.

